# Pearls and Pitfalls for the Direct Posterior Approach to the Proximal Tibia and Knee Joint: A Clinical Experience From a Tertiary Center in Central India

**DOI:** 10.7759/cureus.103474

**Published:** 2026-02-12

**Authors:** Rameez Ahmedkhan R Pathan, Sandesh Subhash Agrawal, Deeptiman James, Shahzad Khan, Devendra Kumar Borker, Dikshant Sharma

**Affiliations:** 1 Orthopaedics, Shri Balaji Institute of Medical Science, Raipur, IND; 2 Orthopaedics, Hindu Rao Hospital and North Delhi Municipal Corporation (NDMC) Medical College, Delhi, IND; 3 Orthopaedics, SPARSH Hospital, Infantry Road, Bangalore, IND; 4 Orthopaedics, Manipal Academy of Higher Education, Mangalore, IND; 5 Spine Surgery, Shree Narayana Hospital, Raipur, IND; 6 Orthopaedics, Sri Devaraj URS Academy of Higher Education and Research Centre, Kolar, IND; 7 Spine Surgery, Stavya Spine Hospital and Research Institute, Ahmedabad, IND; 8 Orthopaedics, Christian Medical College, Vellore, Vellore, IND

**Keywords:** direct posterior approach, neurovascular safety, popliteal cyst, posterior tibial condyle fracture, proximal tibia

## Abstract

Background

The posterior approach to the proximal tibia provides direct access to posterior knee and tibial pathologies, allowing controlled identification and protection of neurovascular structures within the popliteal fossa. Despite these advantages, concerns regarding prone positioning, potential neurovascular injury, wound complications, and limited surgeon familiarity have restricted its widespread use. This study aimed to evaluate indications, perioperative complications, and functional outcomes of the posterior approach to the proximal tibia, highlighting practical pearls and pitfalls.

Methods

A retrospective review was conducted of 21 patients who underwent surgery using a posterior approach to the proximal tibia between March 2021 and December 2024 at Shri Balaji Institute of Medical Sciences, Raipur, a tertiary care center in Central India. Indications included posterior tibial condyle open reduction and internal fixation or posterior cruciate ligament avulsion fixation in nine of 21 patients (42.9%), popliteal cyst excision in five (23.8%), posterior tibial exostosis with impingement in four (19.0%), and excision or curettage of proximal tibial lesions in three (14.3%). All procedures were performed in the prone position using either a direct posterior or posteromedial approach based on lesion location. Operative duration, complications (graded according to the Clavien-Dindo classification), and functional outcomes were assessed using the Lysholm score.

Results

The cohort comprised 21 patients (13 males (61.9%) and eight females (38.1%)) with a mean age of 37.4 years (range, 19-62 years). Two of 21 patients (9.5%) developed Clavien-Dindo grade II complications, including one superficial surgical site infection and one transient tibial nerve neuropraxia, both managed conservatively. No vascular injuries, deep infections, anesthesia-related events, or permanent neurological deficits were observed. At a mean follow-up of 12.6 months, three of 21 patients (14.3%) were lost to follow-up. Among the remaining 18 patients, 17 (94.4%) achieved satisfactory functional outcomes, with a mean Lysholm score of 88.4 ± 5.7, and most returned to their preoperative activity levels.

Conclusions

The posterior approach to the proximal tibia was safe and effective in this cohort, with a low complication rate and favourable functional outcomes. Careful patient selection, meticulous dissection, approach customization based on lesion location, gentle neurovascular handling, maintenance of knee flexion in the prone position, thorough preoperative imaging, and appropriate anesthetic optimization are essential to minimize complications.

## Introduction

The proximal tibia is a complex anatomical region, with anterior, posterior, and lateral columns bordered by critical neurovascular structures in the popliteal fossa. Standard surgical access is commonly achieved via anterolateral or anteromedial approaches, which provide safe exposure to the subcutaneous proximal tibia while minimizing the risk to neurovascular structures [1,2]. However, these approaches offer limited visualization of the posterior tibial plateau, posterior column fractures, and posteriorly located lesions [3,4].

The posterior approach to the proximal tibia, including posteromedial and posterolateral modifications, enables direct visualization and manipulation of posteriorly located fracture fragments, cysts, or tumors while allowing careful protection of the popliteal neurovascular bundle [5-7]. Despite its advantages, this approach is underutilized, likely due to concerns regarding prone positioning, anesthetic challenges, potential neurovascular injury, and surgeon unfamiliarity [6,8].

Several authors have advocated for the posterior approach in selected clinical scenarios. Lin et al. demonstrated improved outcomes for complex posterior column tibial plateau fractures using a direct posterior approach [6], while Cho et al. reported successful management of benign and aggressive proximal tibial tumors via posterior access [9]. Systematic reviews have highlighted that although the posterior approach carries a slightly higher risk of perioperative complications than anterior approaches, meticulous surgical planning and adherence to anatomical planes can minimize these risks [8,10].

The primary objective of this study was to evaluate the safety and functional outcomes of the posterior approach to the proximal tibia. Secondary objectives included identification of surgical indications, perioperative complications, and technical considerations, including practical pearls and pitfalls, to guide safe implementation of this approach.

## Materials and methods

Study design and ethical approval

A single-center, retrospective observational study was conducted at Shri Balaji Institute of Medical Sciences, Raipur, a tertiary referral institution in Central India, with Institutional Ethics Committee (IEC) approval (approval number: SBIMS/IEC/Certi./149/2025). The study adhered to the ethical principles outlined in the Declaration of Helsinki.

Study population

All adult patients who underwent surgery using a direct posterior approach to the proximal tibia at our institution between March 2021 and December 2024 were included in the audit. A total of 21 patients met the study criteria and were included in the analysis.

Inclusion and exclusion criteria

**Figure 1:**
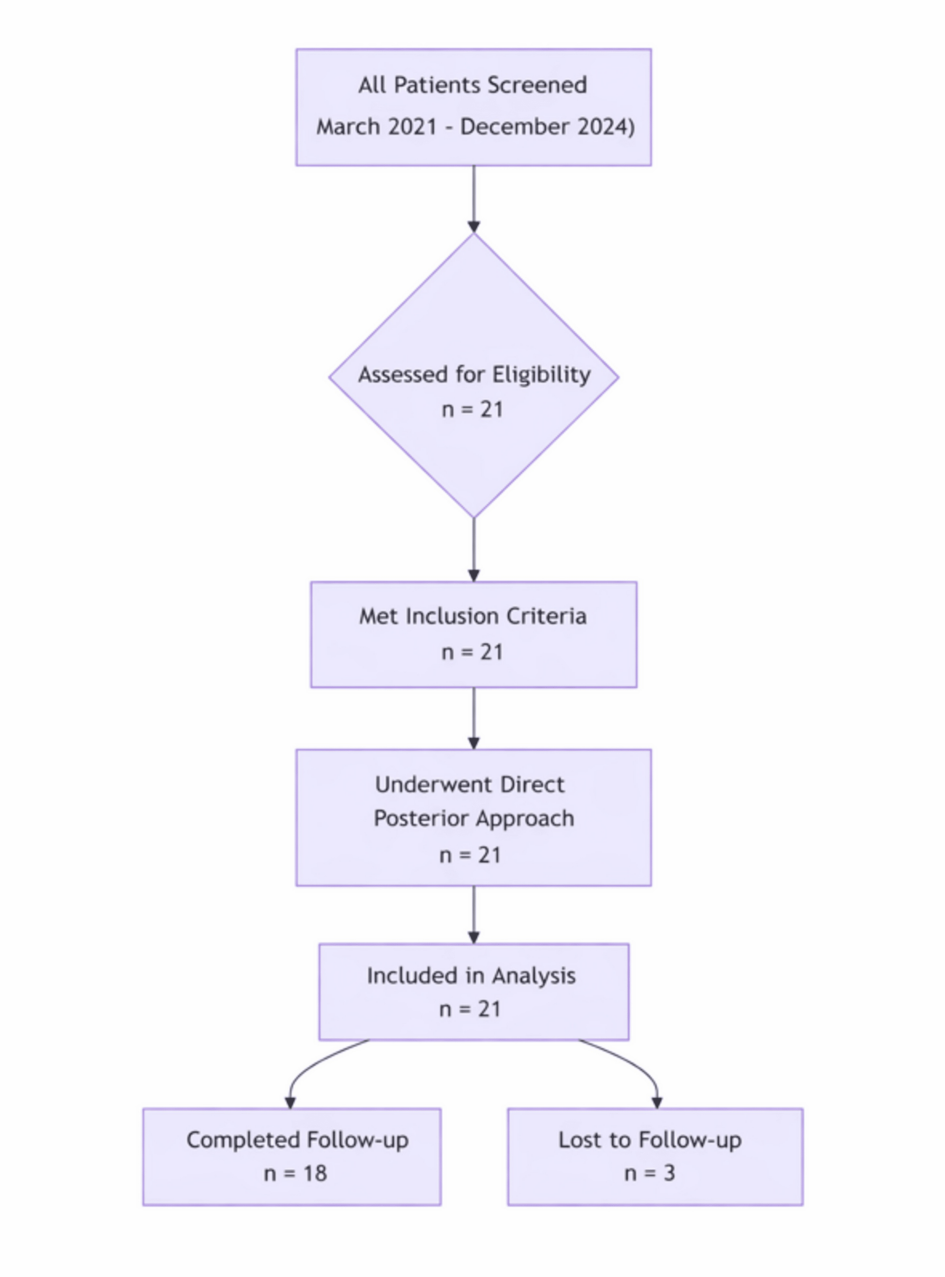
Flowchart

Patients aged 18 years or older with fractures or lesions located on the posterior aspect of the proximal tibia who underwent surgical management through a direct posterior approach and had a minimum follow-up duration of six months were included in the study. Patients were excluded if they presented with open injuries, gross local or systemic infection, pre-existing vascular compromise around the knee, or a history of prior surgery involving the knee that disrupted the posterior anatomical planes. Patient recruitment and selection are summarized in the study, as shown in Figure 1.

Surgical technique

Patient Positioning and Anaesthesia

All procedures were performed with the patient in the prone position under either spinal anaesthesia (SA) or general anaesthesia (GA). Adequate padding was provided over all pressure points, including the shoulders and anterior superior iliac spines, with appropriate chest and pelvic bolsters to maintain abdominal free-hanging and prevent venous congestion.

From an anesthetic standpoint, prone positioning necessitated careful pre-positioning planning. In patients receiving GA, endotracheal intubation was secured prior to turning the patient prone, with strict attention to maintaining neutral cervical spine alignment to avoid cervical cord or vertebral artery compromise. The head was supported in a padded headrest or foam cushion to prevent direct ocular pressure and ensure unobstructed venous drainage, thereby minimizing the risk of postoperative visual complications. Thoracoabdominal compression was avoided to preserve diaphragmatic excursion, venous return, and cardiopulmonary stability. In cases performed under SA, spontaneous ventilation was preserved; however, careful assessment of dermatomal spread was required to ensure adequate anaesthesia for posterior tibial exposure. Hemodynamic variations associated with prone positioning and pneumatic tourniquet use were closely monitored. Continuous communication between the surgical and anaesthesia teams was maintained throughout the procedure to ensure patient safety.

Limb Positioning and Preparation

The operative knee was maintained in approximately 10° of flexion to minimize tension on the sciatic nerve. A pneumatic tourniquet was applied to the proximal thigh prior to sterile preparation and draping. Care was taken to avoid hip hyperextension during positioning, and knee flexion was maintained throughout draping to reduce neurovascular stretch.

Surgical Exposure

**Figure 2:**
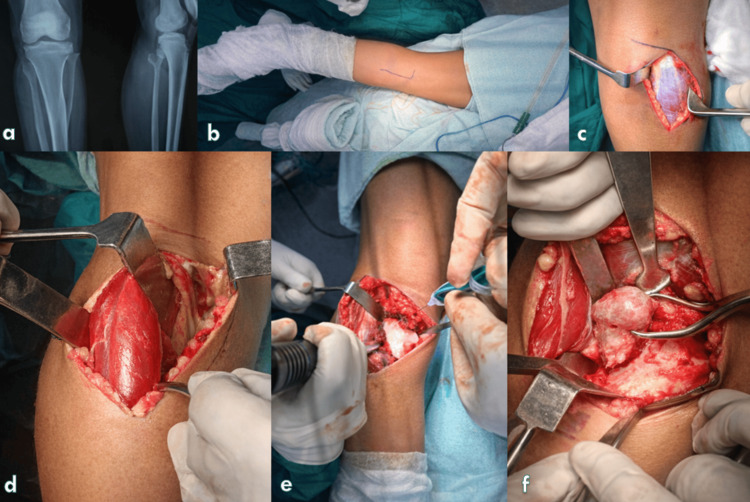
Stepwise surgical exposure of the posterior proximal tibia demonstrating posterior approach and muscle retraction (a) Preoperative anteroposterior and lateral radiographs of the knee. (b) Patient positioning and surface marking of the planned incision along the posterior calf. (c) Skin incision with initial exposure of the deep fascia. (d) Medial head of the gastrocnemius identified and retracted to create a safe posterior corridor. (e) Deep dissection with visualization of the posterior tibial surface and operative field preparation. (f) Final intraoperative exposure demonstrating adequate access to the target region.

The choice of skin incision was determined by the surgeon’s preference and the extent of exposure required, based on preoperative radiographs and MRI or CT imaging. Either a reversed L-shaped incision (Figure 2a) or a lazy S-shaped incision (Figure 2b) was utilized. The longitudinal limb of the incision was placed on the posteromedial or posterolateral aspect of the proximal calf, depending on lesion location.

Full-thickness skin flaps were elevated, and the deep fascia was incised longitudinally. Care was taken to identify and preserve the confluence of the medial and lateral sural cutaneous nerve branches and the sural nerve. When a posteromedial longitudinal incision was used, the subcutaneous saphenous nerve was meticulously preserved. A surgical plane was developed between the pes anserinus and the lateral border of the medial head of the gastrocnemius using blunt and sharp dissection.

Neurovascular Handling and Deep Dissection

The popliteal neurovascular bundle lies deep to the gastrocnemius muscle, with arterial trifurcation typically occurring at the level of the soleus origin along the soleal line. To enhance exposure, the medial head of the gastrocnemius was detached when necessary, allowing the muscle belly to be reflected along with the neurovascular bundle under direct visualization, thereby minimizing the risk of inadvertent injury (Figure 2c). A subperiosteal dissection was then performed to elevate the medial gastrocnemius together with the soleus muscle, providing excellent exposure of the posterior knee joint and proximal tibia (Figure 2d). For lesions located on the posterolateral aspect of the proximal tibia, the posterolateral longitudinal limb of the incision was used to elevate the lateral head of the gastrocnemius, facilitating access to the targeted pathology (Figure 2e).

Closure and Postoperative Protocol

Meticulous haemostasis was achieved, and the wound was closed in layers over a suction drain. A compression dressing and posterior knee splint were applied postoperatively and maintained for two weeks. Thereafter, active-assisted knee range-of-motion exercises were initiated. Progression to weight-bearing was individualized based on the underlying pathology and stability of fixation.

Outcome Measures

Postoperative complications were recorded and graded according to the Clavien-Dindo classification system [11]. This system categorizes complications as follows: Grade I: Any deviation from normal postoperative course without pharmacological or surgical intervention (e.g., mild nausea, transient pain). Grade II: Complications requiring pharmacological treatment (e.g., antibiotics, blood transfusion). Grade III: Complications requiring surgical, endoscopic, or radiologic intervention. Grade IV: Life-threatening complications requiring intensive care unit management. Grade V: Death of the patient.

Functional outcomes were assessed using the Lysholm Knee Scoring System [12]. The score comprises eight domains: limp, support, locking, instability, pain, swelling, stair climbing, and squatting, with a total score ranging from 0 (worst) to 100 (best). Scores were interpreted as follows: excellent (95-100), good (84-94), fair (65-83), and poor (<65). Patients were followed up at two weeks, six weeks, three months, six months, and 12 months postoperatively. Functional outcomes were assessed at each visit using the Lysholm Knee Score, and radiographs were obtained at six weeks, three months, and six months to monitor fracture healing

Statistical Analysis

Descriptive statistics were used to summarize demographic data, surgical indications, and outcomes. Spearman’s correlation coefficient was calculated to assess relationships between surgical duration, complication occurrence, and functional outcomes. A p-value < 0.05 was considered statistically significant. Data are presented as mean ± SD or number (%) Statistical analysis was performed using appropriate standard statistical methods. All analyses were performed using IBM SPSS Statistics for Windows, Version 25.0 (released 2017, IBM Corp., Armonk, NY).

## Results

A total of 21 patients were included in the study. The mean age of the cohort was 37.4 ± 11.2 years (range: 19-62 years). There were 13 males and eight females, yielding a male-to-female ratio of 13:8. The mean follow-up duration was 12.6 ± 4.3 months. Three patients (14.3%) were lost to follow-up. Baseline demographic and clinical characteristics are summarized in Table 1.

**Table 1:** Baseline demographic and clinical characteristics Legend: Values are presented as mean ± standard deviation or number (%). SD: standard deviation; n: number of patients. Three patients were lost to follow-up.

S.no	Characteristic	Value
1.	Total patients	21
2.	Male: Female	13: 08
3.	Mean age (years)	37.4 ± 11.2
4.	Mean follow-up (months)	12.6 ± 4.3
5.	Patients lost to follow-up	3 (14.3%)

Surgical indications and incision types

The surgical indications varied across the cohort. Posterior tibial condyle fractures with or without associated posterior cruciate ligament (PCL) avulsion were the most common indication, accounting for nine cases (42.9%). Popliteal cyst excision was performed in five patients (23.8%), followed by surgery for symptomatic posterior tibial exostosis in four patients (19.0%). Excision or curettage of proximal tibial lesions was undertaken in three patients (14.3%).

The choice of skin incision was determined by the underlying pathology and the need for surgical exposure. A reversed L-shaped incision was used in 11 patients, and a lazy S-shaped incision in 10. The distribution of surgical indications and corresponding incision types is detailed in Table 2.

**Table 2:** Distribution of surgical indications and corresponding incision types The table shows the number and percentage of patients for each indication, along with the type of posterior incision utilized: reversed L-shaped or lazy-S. n: number of patients; %: percentage of the total cohort.

S. no.	Surgical indication	n (%)	Primary incision type
1.	Posterior tibial condyle and PCL avulsion fractures	9 (42.9%)	Reversed L-shaped (7/9)
2.	Popliteal cyst excision	5 (23.8%)	Lazy S-shaped (4/5)
3.	Symptomatic posterior tibial exostosis	4 (19.0%)	Reversed L-shaped (3/4)
4.	Proximal tibial lesion excision/curettage	3 (14.3%)	Lazy S-shaped (2/3)
	Total	21 (100%)	Reversed L: 11, Lazy S: 10

Complications and functional outcomes

Postoperative complications were infrequent. Two patients (9.5%) experienced complications classified as Clavien-Dindo grade II. One patient developed a superficial surgical site infection, which was successfully managed with oral antibiotics and local wound care. Another patient experienced transient tibial nerve neuropraxia, which resolved completely within six weeks. There were no cases of deep infection, vascular injury, or permanent neurological deficit.

Functional outcomes were favourable in the majority of patients. The mean Lysholm Knee Score at final follow-up was 88.4 ± 5.7, with 19 of 21 patients (90.4%) achieving good-to-excellent outcomes. All patients who underwent fracture fixation demonstrated radiological union, with a mean time to union of 13.6 ± 1.2 weeks (range: 12-16 weeks). The mean postoperative knee range of motion was 0° to 120°. A summary of complications and functional outcomes is provided in Table 3 .

**Table 3:** Complications and functional outcomes Complications are classified according to the Clavien-Dindo grading system. Functional outcomes were assessed using the Lysholm Knee Score, reported as mean ± SD. ROM: range of motion; SSI: surgical site infection; n: number of patients.

S. no.	Outcome parameter	Value
01	Overall complication rate	2/21 (9.5%)
02	Clavien–Dindo grade II	2/21 (9.5%)
03	Superficial surgical site infection	1 (4.8%)
04	Transient tibial neuropraxia	1 (4.8%)
05	Major complications	0
06	Mean Lysholm Knee Score	88.4 ± 5.7
07	Good-to-excellent outcomes	19/21 (90.4%)
08	Mean time to union (weeks)	13.6 ± 1.2
09	Mean knee range of motion	0 –120°

Correlation analysis

Spearman’s correlation analysis demonstrated a weak inverse relationship between surgical duration and Lysholm Knee Score (n = 18; r = −0.32, p > 0.05), suggesting slightly lower functional scores with increasing operative time. A moderate positive correlation was observed between complication occurrence and surgical duration (n = 21; r = 0.41, p > 0.05), indicating a trend toward a higher likelihood of complications with prolonged surgery. However, neither association reached statistical significance.

## Discussion

**Figure 3:**
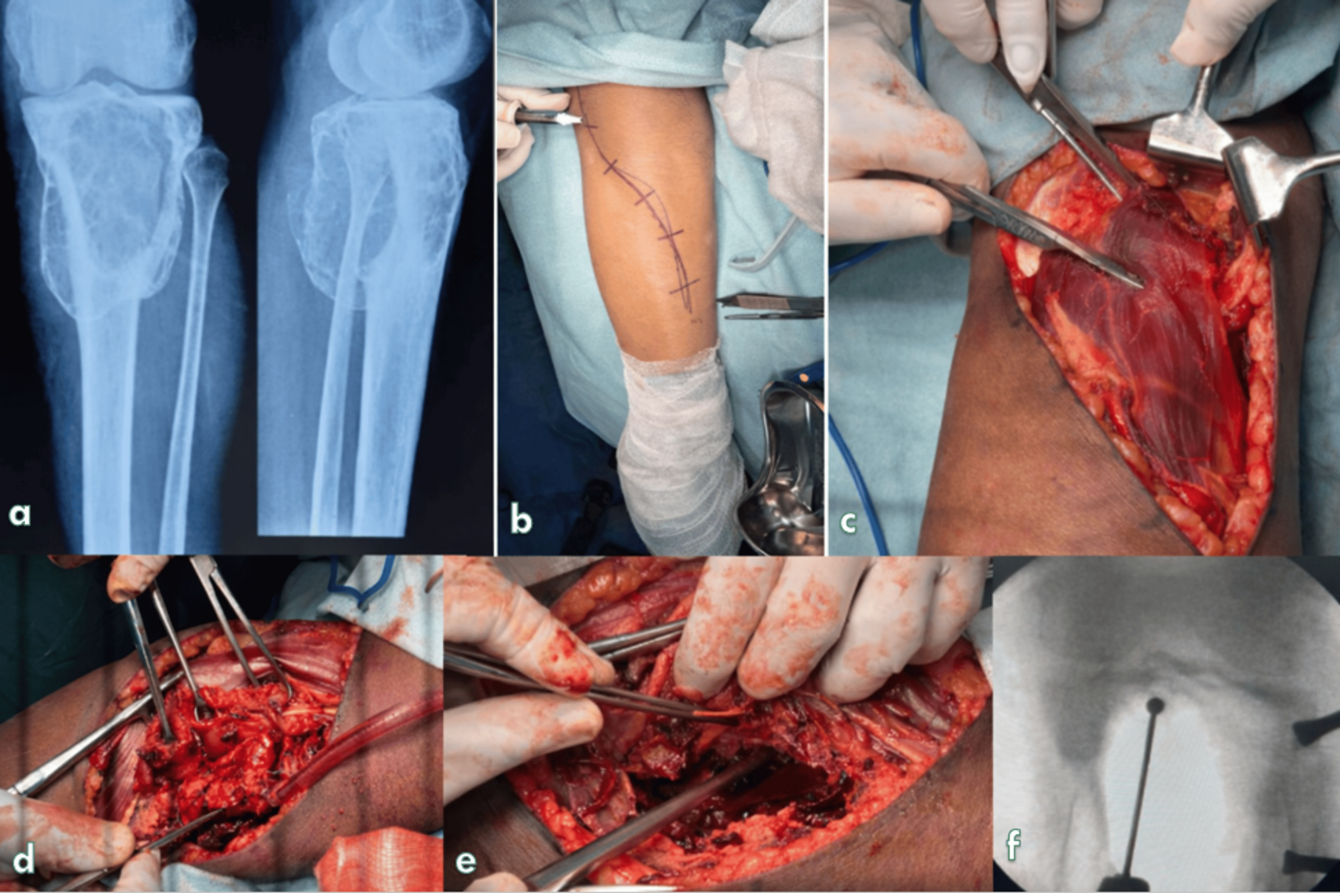
Representative preoperative radiographs and intraoperative surgical steps from patients in the present study demonstrating the direct posterior approach (a) Preoperative radiographs showing the involved knee. (b) Surgical site preparation with incision planning and anatomical landmarks marked. (c) Superficial and deep soft-tissue dissection with protection of surrounding structures. (d) Progressive muscle retraction and development of the posterior interval. (e) Direct visualization and handling of the lesion/target site during definitive procedure. (f) Intraoperative fluoroscopy confirming instrument/implant positioning.

**Figure 4:**
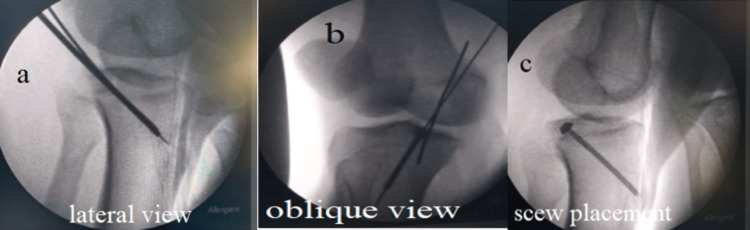
Intraoperative fluoroscopic images from a patient in the present study demonstrating guidewire trajectory and screw placement Fluoroscopic views obtained during surgery in a study patient showing (a) lateral view confirming guidewire position, (b) oblique view demonstrating trajectory planning, and (c) final screw placement with satisfactory alignment.

The surgical anatomy of the proximal tibia is largely defined by the relationship of the anterior, posterior, and lateral neurovascular structures. Owing to the subcutaneous location of the anteromedial and anterolateral surfaces of the proximal tibia, anterior approaches are most commonly employed, as they allow relatively safe and straightforward access while avoiding the popliteal neurovascular bundle [1,4]. Nonetheless, anterior approaches may not adequately visualize posterior column fractures or lesions, potentially compromising the accuracy of reduction and the stability of fixation in some patients [5,6]. As demonstrated in Figures 3-4, careful creation of the posterior interval provided a well-defined and protected operative corridor, minimizing neurovascular risk while enabling secure lesion access and intraoperative fluoroscopic guidance.

The direct posterior and posteromedial approaches, although less frequently utilized, offer excellent exposure of the posterior columns of the proximal tibia and the posterior aspect of the knee joint. These approaches facilitate controlled dissection, direct visualization, and judicious retraction of the popliteal neurovascular structures, thereby enabling safe placement of anteriorly directed fixation constructs [6-9]. Although posterior approaches offer several advantages, they are often avoided due to the anesthetic complexities of prone positioning and concerns about accidental injury to the popliteal vessels or tibial nerve [1,2].

The posterolateral approach, in particular, has been associated with an increased risk of injury to the common peroneal nerve due to its anatomical proximity [2,13]. Excessive or forceful retraction during posterior exposure may also result in tibial nerve neuropraxia or injury to the popliteal vessels and their branches [8]. Inadequate hemostasis in the richly vascularized posterior knee region can lead to postoperative hematoma formation and elevated compartment pressures. Therefore, meticulous dissection, gentle tissue handling, and thorough hemostasis are essential prerequisites for the posterior approach, underscoring the importance of careful preoperative planning and the surgeon's familiarity with posterior knee anatomy.

Several authors have advocated the use of direct posterior or modified posterior approaches to improve access and outcomes in complex posterior tibial plateau fractures. Lin et al., Chouhan et al., and Hake et al. have demonstrated that posterior-based approaches allow superior visualization and anatomical reduction of posterior column fractures compared to traditional anterior approaches [6,7,9]. Kottmeier et al. recommended the posterior approach for select medial tibial plateau fractures, particularly when posterior fragment fixation is required [8]. Nicandri et al. described a modified posterior approach for fixation of posterior cruciate ligament (PCL) avulsion fractures, although arthroscopic fixation has since become the preferred modality for many such injuries [10].

The posterior approach is not limited to trauma-related pathology. Cho et al. reported its successful use in managing benign and aggressive bony and soft-tissue tumors arising from the proximal tibia, highlighting its utility beyond fracture fixation [13]. In our series, indications for the posterior approach were almost equally distributed between traumatic posterior column fractures and posteriorly located bony or soft-tissue lesions. In addition to four patients with large posterior tibial exostoses causing impingement symptoms, three patients with epimetaphyseal expansile lytic lesions (giant cell tumor, n = 1; aneurysmal bone cyst, n = 2) underwent extended curettage and bone grafting. The predominant involvement of the posterior cortex necessitated a direct posterior approach, which allowed adequate tumor clearance while preserving the integrity of the anterior and mediolateral cortices.

The versatility of the posterior approach is further enhanced by modifications of the distal longitudinal limb of the incision, which can be extended medially or laterally depending on lesion location and surgical requirements [7,14]. Proximal extension of the incision improves access to the posterior knee joint and distal femur while reducing retraction pressure on critical neurovascular structures. In our cohort, a posteromedial approach was used in 13 patients and a posterolateral approach in the remaining cases. None of the patients required a dual-incision strategy, reflecting the adequacy of preoperative imaging-based planning.

The posterior approach is generally contraindicated in polytrauma patients and in those with associated thoracoabdominal injuries or significant medical comorbidities that render prone positioning unsafe [1,15]. Consequently, polytrauma patients and those with suspected vascular injuries-where vascular exploration takes precedence-were excluded from our study. Systematic reviews comparing anterior and posterior approaches to the proximal tibia have reported a slightly higher rate of perioperative complications with posterior approaches [1,15]. Factors contributing to this include surgeon unfamiliarity, gravity-dependent wound positioning, prolonged operative time, and challenges in postoperative wound care.

Reported complications associated with posterior approaches include superficial wound infections, wound dehiscence, hardware irritation, neuropraxia, and vascular injury [16-17]. In our series, one patient developed a superficial surgical site infection that resolved with extended antibiotic therapy and local wound care, and another experienced transient common peroneal nerve neuropraxia with foot drop, which resolved completely. Both complications were classified as Clavien-Dindo grade II, consistent with prior reports [16,17]. Importantly, no major neurovascular injuries or deep infections were observed, supporting the safety of the posterior approach when performed with appropriate precautions.

This study has several limitations. Its retrospective design and small cohort size inherently introduce methodological heterogeneity, including variability in surgeon judgment and patient characteristics. The single-center setting and heterogeneous patient indications limit the generalizability of our findings. The descriptive nature of the study precludes causal inferences, and subgroup or comparative analyses by indication were not feasible due to the small sample size. In addition, the follow-up duration, while adequate for short-term functional assessment, may not fully capture long-term outcomes or potential late complications. These factors should be considered when interpreting the results, particularly regarding functional outcomes and complication rates.

## Conclusions

The posterior approach to the proximal tibia was found to be a reliable and practical surgical option for accessing posteriorly located proximal tibial pathology, including traumatic fractures and benign tumor excision. Although traditionally underutilized due to concerns regarding neurovascular injury and prone positioning, no major complications were observed in our series. With meticulous surgical technique, the surgical planes between the medial gastrocnemius and semimembranosus for the posteromedial approach, and between the popliteus and lateral gastrocnemius for the posterolateral approach, provided consistent and safe access to the posterior proximal tibia.

This approach allowed direct visualization and manipulation of posterior fracture fragments, facilitated excision of posteriorly located cysts and masses, and obviated the need for extensive anterior soft-tissue retraction. Our findings are consistent with previously published studies which support the posterior approach as a viable and effective alternative for selected posterior proximal tibial pathologies.
